# A density-based threshold model for evaluating the separation of particles in heterogeneous mixtures with curvilinear microfluidic channels

**DOI:** 10.1038/s41598-020-75878-w

**Published:** 2020-11-04

**Authors:** Chun Kwan Chen, Bee Luan Khoo

**Affiliations:** Department of Biomedical Engineering, City University of Hong Kong, 83 Tat Chee Avenue, Kowloon, Hong Kong, China

**Keywords:** Biomedical engineering, Lab-on-a-chip

## Abstract

Particle separation techniques play an important role in biomedical research. Inertial focusing based microfluidics using nonlinear channels is one of the promising label-free technologies for biological applications. The particle separation is achieved as a result of the combination of inertial lift force (*F*_*L*_) and Dean drag force (*F*_*D*_). Although the mathematical expressions of *F*_*L*_ and *F*_*D*_ have been well derived in prior studies, they are still complicated, which limits their popularity in practice. Recent studies modified these expressions through experiments and proposed a threshold model, which assumes that only particles larger than the threshold will be well focused. Although this threshold model has been used in recent studies, two varying versions of the threshold model (TM1 and TM2) prevents standardisation in practice. In addition, both models were developed with regular low-density particles and may not be applicable to samples with higher density or samples with irregular shapes. Here, we evaluated the threshold models with samples of different densities. Based on these evaluations, we derived a modified model (TM4), which additionally considers the factor of particle density to improve the accuracy of existing models. Our results demonstrated that TM4 could more reliably predict the sorting efficiency of samples within a wider density range.

## Introduction

Microfluidic technology is the design and study of microdevices with microscale channels that can analyze and process small amounts of fluids^1^. The use of microchannels has many advantages, including low cost, fast processing time, less sample consumption, and sample detection capabilities with high sensitivity and specificity^1,2^. Because of these benefits, microfluidic technology has been widely used in biomedical applications, such as the separation of biological samples^2^.

Conventional antibody-based biological sample separation methods can achieve high specificity and sensitivity, but they require high antibody costs and can damage or contaminate the target during the labelling process^3,4^. Specifically, the immobilization of proteins preserves the cellular structure but may interfere with epitope recognition. In addition, for antibodies that label cell surface membrane proteins, protein fusion can also modify protein behaviour^5^. Therefore, many label-free microfluidic methods have been developed to separate particles based on differences in the inherent physical properties (such as size, density, and deformability)^4,6^. Label-free microfluidic techniques for particle separation can be further classified as active and passive approaches. Active approaches, such as acoustophoresis and dielectrophoresis, utilize external fields, while passive approaches, such as deterministic lateral displacement (DLD) and pinched flow fractionation (PFF), capitalise on hydrodynamic forces and channel geometry instead of external fields. Despite advances in these technologies, inherent problems continue to hinder their widespread application (Table S1). Typically, active devices can achieve higher separation efficiency, but due to the complicated channel design of the external fields, the device throughput is usually reduced and associated with high costs^7^. Passive devices using DLD or PFF provide excellent resolution in particle separation, but technical challenges such as the requirements of narrow channels can influence particle interactions and promote channel fouling, resulting in reduced flux^8,9^.

In the past decade, inertial microfluidic technology has attracted widespread research interest due to its high-throughput, simple, and label-free operation^10–12^. In the microfluidic system, the inertial effect becomes significant in the Reynolds number (*Re*) range of 1 to 100^12^. In curvilinear channels, the microscopic inertial focusing is mainly caused by two forces, inertial lift force (*F*_*L*_) and Dean drag force (*F*_*D*_)^10^. The *F*_*L*_ is the balance of the shear gradient force and the wall induction force acting on the particles to migrate across the streamline to an equilibrium position between the centerline and the channel wall. The radial pressure gradient generated by the centrifugal force acting on particles migrating in the curved channel will result in two counter-rotating vortices, where F_D_ is the two counter-rotating vortices at the cross-section of the curvilinear channel generated by the higher momentum of the flowing fluid near the center, which induces a drag force acting on the particles^11^.

Since the *F*_*L*_ and *F*_*D*_ are size-depending and the degree of *F*_*L*_ experienced by particles also depends on the lateral position, particles with different sizes will experience different degrees of *F*_*L*_ to balance *F*_*D*_ at different lateral positions, resulting in a size-based separation^12^. For example, larger particles will encounter a larger *F*_*L*_ to balance *F*_*D*_ at the position closer to the inner channel wall compared to smaller particles. The utilization of this phenomenon leads to particle separation based on particle size. Although curvilinear microfluidic channels with rectangular cross-sections have been widely used, other channels with varied geometric designs (e.g., channels with trapezoidal cross-sections)^10,12^ have recently become more prevalent due to higher separation resolution in specific applications^13,14^.

Many numerical methods for predicting particle motion in inertial focusing systems have been proposed in the past few years. Examples of these numerical techniques include Asymptotic analysis, Navier–Stokes-based solution, and Lattice Boltzmann method (LBM)^15^. Briefly, the asymptotic analysis estimates the inertial forces acting on the particles by simplifying the fluid equation. Over-simplification often leads to unreliable results in practice^15^. The Navier–Stokes-based solution greatly resolved the drawbacks of asymptotic analysis, such as particle effects on fluid streamlines, but this technique requires a high computing power (time) to solve Eqs. ^16^. Compared with the Navier–Stokes-based solution, LBM can provide a reliable solution that requires less computing power, thereby gaining popularity over the last decade^17^. However, its accuracy is lower for non-straight geometries (e.g., spiral) due to its cubic lattice nature^15^.

In addition to numerical methods, the utilization of size-dependent characteristics of *F*_*L*_ and *F*_*D*_ is also essential for the design of inertial focusing systems. Although the expression of *F*_*L*_ and *F*_*D*_ was derived mathematically in the early studies, it is too complicated because some parameters vary with particle position and channel curvature^12^. In previous studies, the expression of *F*_*L*_ and *F*_*D*_ was modified, and it was concluded that in order to achieve the focusing effect, the ratio of particle diameter (*a*) to channel hydraulic diameter (*D*) should be equal to or greater than 0.07 ($a/D\ge 0.07)$^18^. This threshold model (defined as TM1) suggests that particles larger than the threshold size will experience a considerable inertial lift force, which will balance the Dean resistance and make them focused, while particles significantly smaller than the threshold will remain unfocused (or weakly focused) to migrate along with Dean vortices^12^. Later studies recommended that for a rectangular section with a high aspect ratio (width > height), the inertial focusing depends on the channel height (*h*) rather than the hydraulic diameter, so TM1 was modified to $a/h\ge 0.07$ (defined as TM2)^7,19^. Although TM2 was proposed after TM1, there were still some studies using TM1 as a focusing criterion^20–22^.

A precise threshold model is very important in biological applications because the deviation of different models will lead to underestimation (or overestimation) of particle threshold, which will lead to lower separation efficiency. In addition, since both models were developed using low-density samples ($\sim 1.05$ g/ml) with regular geometric shapes (spherical), they may not be suitable for samples with various density and geometries.

Taking into account the differences between the two threshold models and their applicability for samples with different densities and geometries, we evaluated the accuracy of the two threshold models for various samples, including regular and non-regular polystyrene (PS) particles, non-regular polyamide (PA), polyethylene terephthalate (PET) and talcum (Talc) particles of different densities and geometries. Using these results, we proposed a threshold model based on the introduction of the density coefficient, which assumed that the density coefficient would increase linearly with the particle density.

## Results

### Design principle of the microfluidic system

In the microfluidic system, inertial force becomes significant when the *Re* is around 1 to 100 (~ 1 < *Re* <  ~ 100), which is given by:

1 $$Re=\frac{\rho UD}{\mu }$$

where ρ is the fluid density, *U* is the maximum fluid velocity, μ is the fluid viscosity, and *D* is the hydraulic diameter expressed as:

2 $$D=\frac{2hw}{h+w}$$

where *h* and *w* are the height and width (for rectangular cross-section), respectively. In inertial microfluidics, the resultant force due to shear-gradient and wall-induced forces acting on particles in the opposite direction is called *F*_*L,*_ which is given by:

3 $${F}_{L}=\frac{{C}_{L}{U}^{2}{a}^{4}}{{D}^{2}}$$

where *C*_*L*_ is lift co-efficient, which is a function of particle position, and *a* is the particle diameter. Apart from inertial lift force, particles flowing in the curved channel will experience a *F*_*D*_ resulting from 2 counter-rotating vortices (Dean vortices), which can be quantified by Dean number (*De*) as:

4 $$De=Re\sqrt{\frac{D}{2R}}$$

where *R* is the radius of curvature of the channel path. The Dean vortices induced at the channel cross-section will cause particles to move back and forth across channel width. This lateral velocity for particles, Dean velocity (*U*_*D*_), is estimated by:

5 $${U}_{D}={1.8\times 10}^{-4}{De}^{1.63}$$

With Dean velocity (*U*_*D*_), the *F*_*D*_ acting on the particles can be expressed as:

6 $${F}_{D}=3\pi {U}_{D}a$$

The focusing effect for given particle dimensions is determined by the ratio of *F*_*L*_ and *F*_*D*_ (*R*_*f*_ = *F*_*L*_*/F*_*D*_), which varies with particle diameter (*R*_*f*_ ∝ *a*^3^). The largest particles will be focused on the channel position closest to the inner wall as they experience the larger *F*_*L*_ compared to other particles at that position in the system. A threshold model was derived based on the modification of previously established Eqs. (3), (4), (5), and (6), among which only particles larger than the threshold will experience significant *F*_*L,*_ thereby balancing *F*_*D*_ to facilitate particle focusing. The threshold of particle focusing is proportional to the size of the microchannel, which can satisfy the following inequality, as stated:

7 $$\frac{a}{x} \ge 0.07$$

where $x=D ($ hydraulic diameter) for TM1 and $x=h$ (channel height) for TM2.

**Figure 1:**
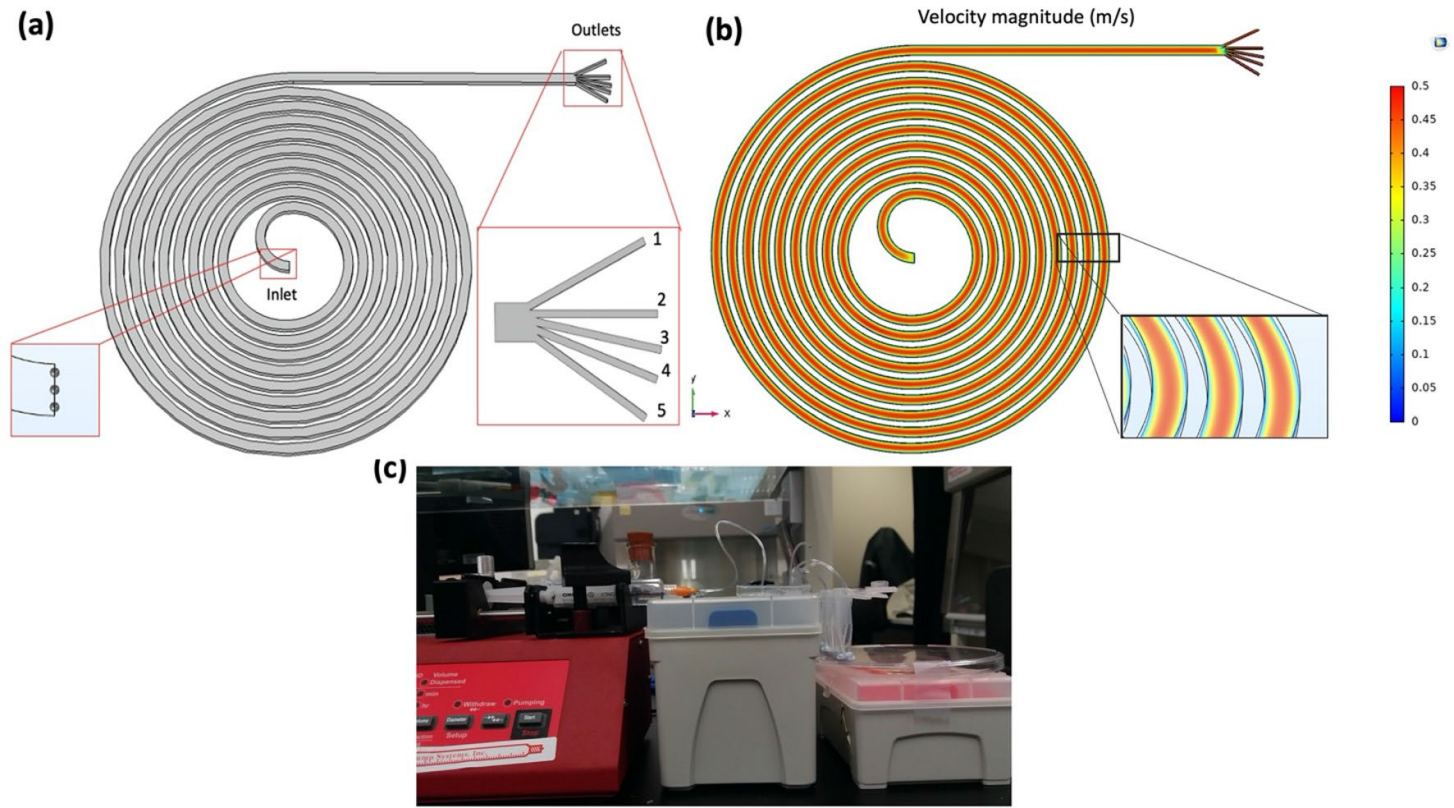
COMSOL flow simulation and experimental setup of the microdevice. (**a**) COMSOL image of the device. The outlets were designated as 1–5. The simulation outcomes of particle motion demonstrated that the particles (set as 1.05 g/ml) were uniformly distributed at the inlet boundary (x–y plane). For clarity, a scale factor was added to the particles. (**b**) COMSOL simulation of a laminar flow profile with a flow velocity of 0.257 m/s at the inlet. (**c**) The experimental setup used for particle sorting experiment.

Particle recovery rate (*PRR*) is often used to quantify the particle sorting performance in microfluidics^23^. In this study, the PRR of the device used (Fig. 1) is defined as:

$$PRR=\frac{Number\,of\,particles\,in\,target\,outlet}{Total\,number\,of\,particles\,in\,all\,outlet}\times 100\%$$

### Evaluation of TM1 and TM2 with regular low-density particles

**Figure 2:**
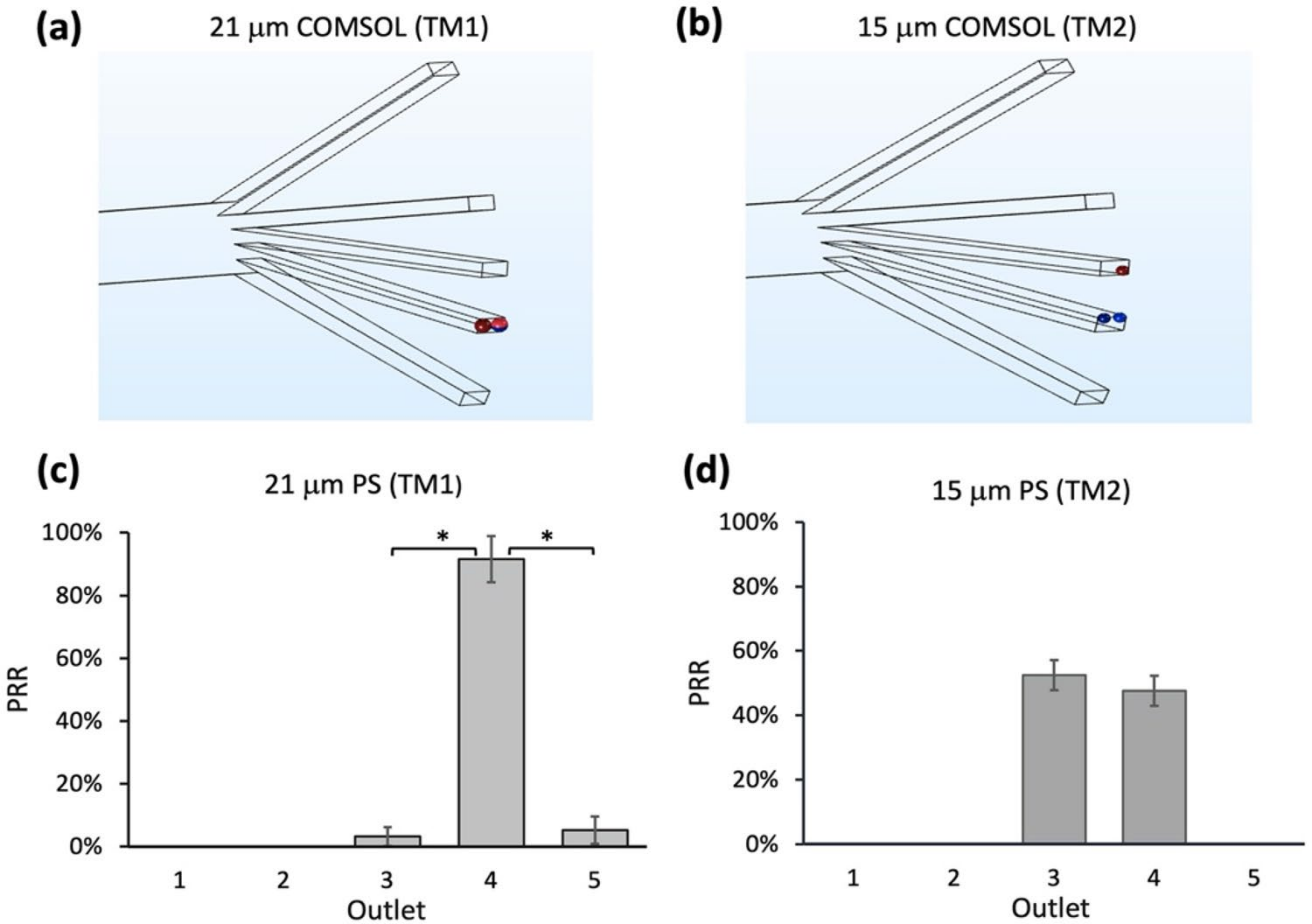
The separation efficiency of 21 μm and 15 μm polystyrene (PS) microspheres, used to evaluate TM1 and TM2 thresholds. COMSOL simulations for (**a**) 21 μm and (**b**) 15 μm particle trajectories that terminated at the respective outlets. For clarity, a scale factor was added to the particles. (**c**) The recovery rate of 21 μm PS microspheres at outlet 4 was 91.6 ± 7.3%. (**d**) The recovery rates of 15 μm PS microspheres were 52.4 ± 4.6% and 47.6 ± 4.7% at outlets 3 and 4, respectively. Data were shown as mean ± SD of three independent experiments; *p < 0.01, **p < 0.001, ***p < 0.001.

Given that TM1 and TM2 were developed with regular low-density particles ($\sim 1.05$ g/ml), both threshold models were initially analysed by COMSOL, and then polystyrene (PS) microsphere experiments were performed. According to Eq. (7), the estimated thresholds of TM1 and TM2 are 21 μm and 15 μm, respectively. Therefore, 21 μm and 15 μm regular PS particles were first studied. In the COMSOL simulation, it was found that 21 μm particles (threshold of TM1) were collected at outlet 4 (Fig. 2a), and 15 μm particles (threshold of TM2) were collected at outlets 3 and 4, respectively (Fig. 2b). Using the estimated values provided by COMSOL, we proceeded to conduct actual experiments to validate this hypothesis further.

For the experiments with 15 μm and 21 μm PS microspheres, more than 90% (91.6 ± 7.3%) of 21 μm particles were recovered in outlet 4 (Fig. 2c), and the rest of the particles was found at outlets 3 and 5 (5.2 ± 3% and 3.2 ± 4.4%, respectively). The particles found in outlets 3 and 5 could be a result of unfocused streamlines in the first few seconds of flow (Fig. S1). For 15 μm particles, the PRRs at outlets 3 and 4 were 52.4 ± 4.6% and 47.6 ± 4.7%, respectively (Fig. 2d). The highest PRR of 21 μm microspheres indicated that the threshold estimated by TM1 for PS microspheres was more accurate than TM2 for devices with similar spiral radius and aspect ratio parameters.

### Evaluating the overestimation of TM1 threshold

The previous results cannot exclude the possibility of overestimating the threshold. For example, even if the threshold in the system was 21 μm (TM1), particles larger than 21 μm (such as 25 μm) would still be well focused, but 25 μm was definitely not the precise threshold value. To address this issue, we studied the PRRs of microspheres in the range of 21 to 15 μm. If the recovery rate of a certain size of particles was very close to the recovery rate of 21 μm particles, TM1 could overestimate the threshold. We defined a systematic method called the 'averaging test' to facilitate this type of analysis process. First, we chose the average value between the TM1 and TM2 thresholds as the testing threshold (T_t_): (21 + 15)/2 = 18 μm. The PRR of 18 μm particles (T_t_ = 18 μm) was then obtained. When there was no significant difference between the highest PRR (hPRR) of 18 μm and the hPRR of 21 μm, a more accurate threshold (T) was present within 15—18 μm (15 μm < T < 18 μm). Consequently, the average value of 15 μm and 18 μm would be taken as the next testing threshold. However, if the hPRR of 18 μm was significantly lower than that of 21 μm PRR, it indicated that the target threshold was between 18 to 21 μm (18 μm < T < 21 μm), and the average value of 18 μm and 21 μm would be taken as the next testing threshold.

**Figure 3:**
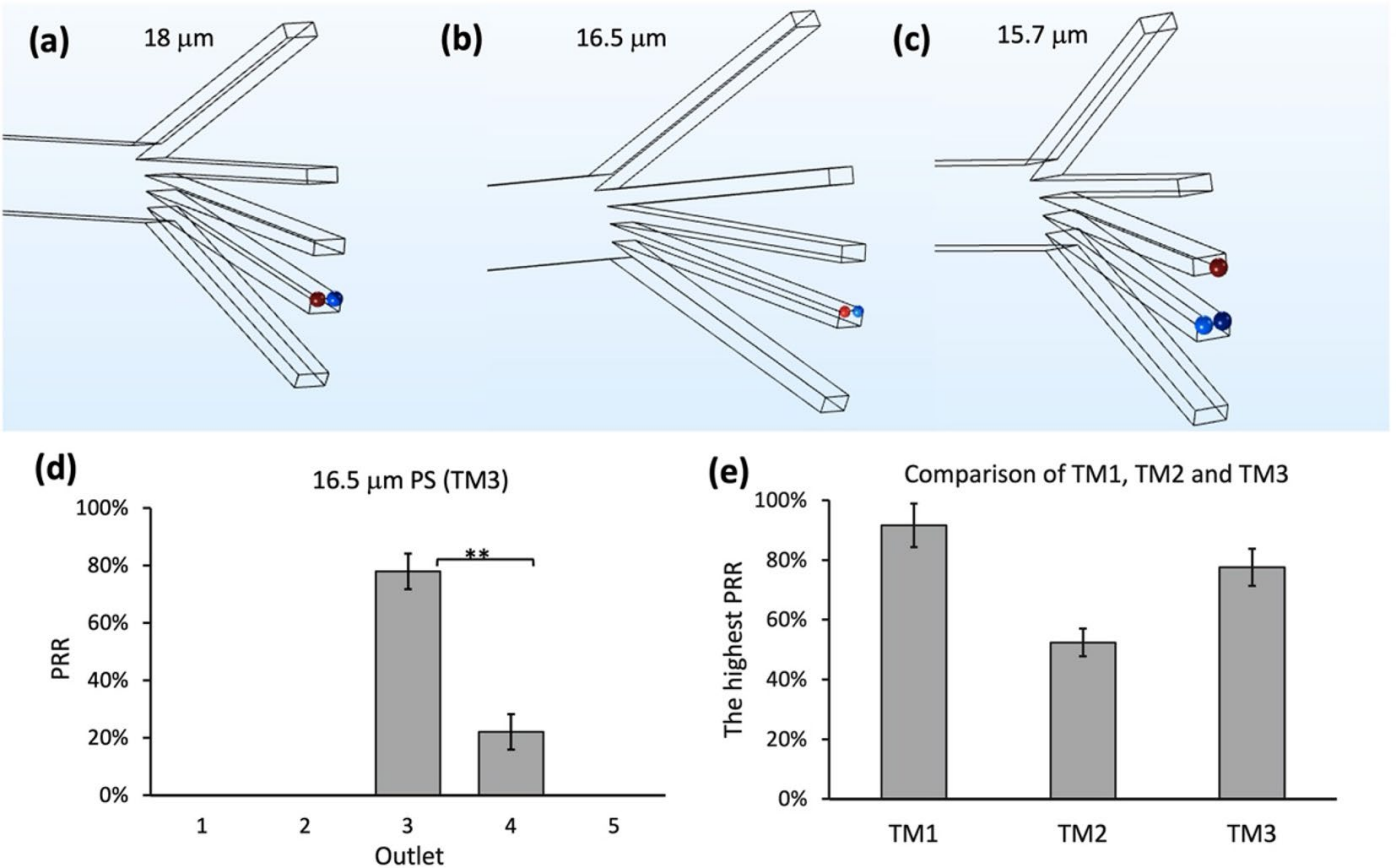
COMSOL simulation of particle trajectories for 18 μm, 16.5 μm and 15.7 μm particles. The recovery rates of (**a**) 18 μm, (**b**) 16.5 μm and (**c**) 15.7 μm particles at their respective outlets. For clarity, a scale factor was added to the particles. (**d**) Experimental results of 16.5 μm PS microspheres. (**e**) Comparison of the highest experimental recovery rates of TM1, TM2, and TM3 for PS microspheres. Data were shown as mean ± SD of three independent experiments. *p < 0.01, **p < 0.001, ***p < 0.0001.

18 μm was taken as the first testing threshold (test 1) (Table S2). All 18 μm particles (T_t1_ = 18 μm) were well focused on outlet 4 (Fig. 3a), which was similar to the PRR of 21 μm particles (Fig. 2a). Hence, the threshold value of 16.5 μm (T_t2_ = (18 + 15)/2 = 16.5 μm) (test 2) was used for the next test. Then, all 16.5 μm particles were well focused on outlet 4 (Fig. 3b), which was similar to the PRR of 18 μm particles (T_t1_). Hence the threshold value of 15.7 μm (T_t3_ = (16.5 + 15)/2 $\sim$ 15.7 μm) was used for the next test (test 3). The simulation of 15.7 μm particles showed that only 66.7% particles (n = 3) were concentrated on outlet 4 (Fig. 3c), significantly lower than the PRR of 16.5 μm particles. As a result, the target threshold range was likely to be between 16.5 μm and 15.7 μm (15.7 μm < T < 16.5 μm), and the theoretical threshold value for test 4 was defined as 16.1 μm. Since the precise threshold range was between 16.1 μm and 16.5 μm (16.1 μm < T < 16.5 μm), the scale of overestimation in practice was negligible. Hence, the revised upper limit of 16.5 μm was used as a proposed threshold for regular low-density samples. For the corresponding threshold model (TM3) modified from TM1 and TM2, the $x$ in Eq. (7) was taken to $(D+3h)/4$.

The outcomes of COMSOL simulation were validated by actual experiments. Specifically, we evaluated TM3 with 16.5 μm PS microspheres (Fig. 3d). The highest PRR was found at outlet 3 (77.6 ± 6.2%), while the rest of the microspheres was found at outlet 4 (22.4% ± 6.2%). Although the hPRR of 16.5 μm microspheres at outlet 3 were higher than that of 15 μm microspheres (52.4 ± 4.6%), it was lower than that of 21 μm microspheres (91.6 ± 7.3%) (Fig. 3e). Thus, TM1 remains the most accurate model for regular low-density particles in this study.

### Evaluation of all models with irregular low-density particles

**Figure 4:**
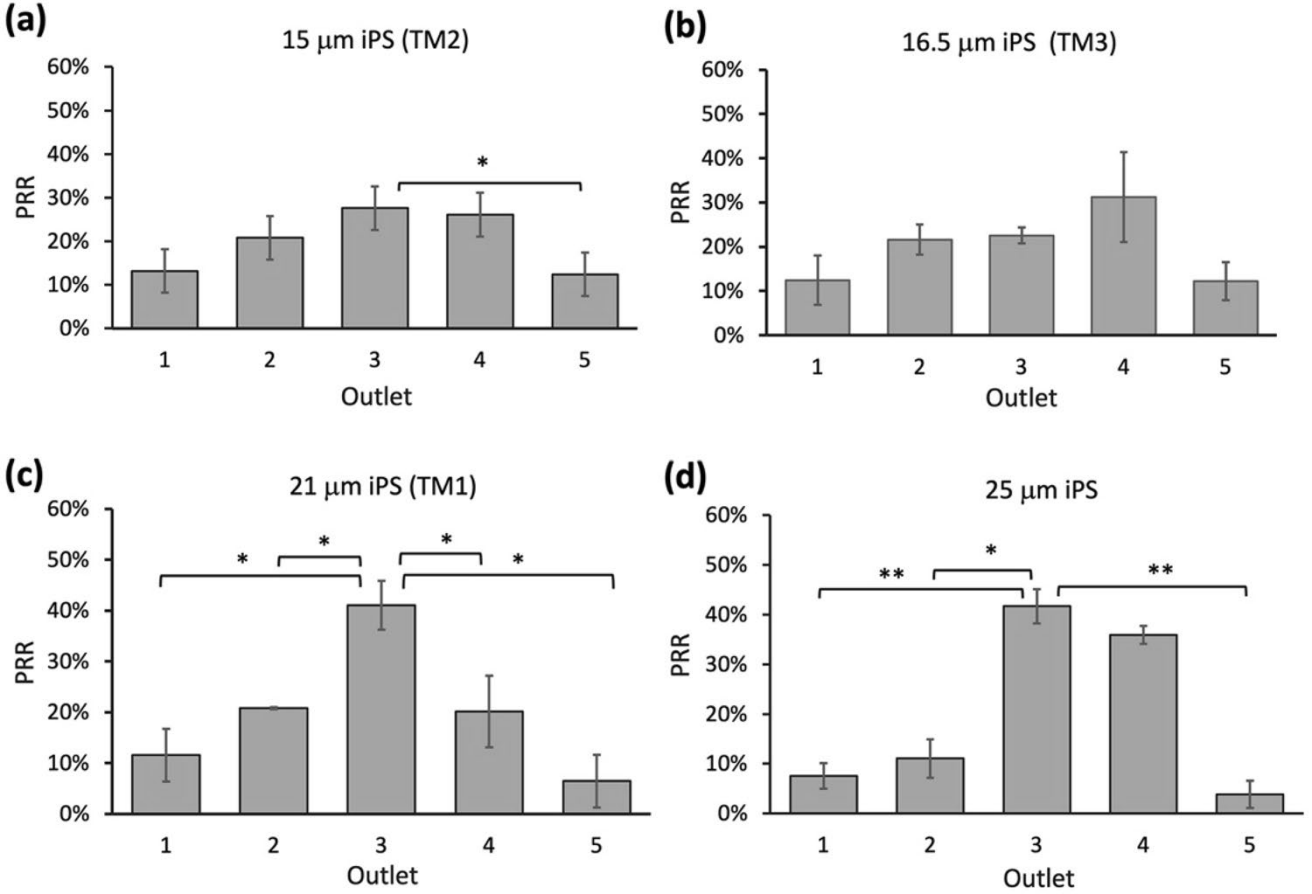
Recovery rate of irregular polystyrene (iPS) particles of 15 μm, (**b**) 16.5 μm, (**c**) 21 μm, and (**d**) 25 μm (± 2 μm) diameters. Since the hPRRs of 21 μm and 25 μm were very close (41% and 41.7%), 21 μm was determined as the threshold for iPS. Data were shown as mean ± SD of three independent experiments. *p < 0.01, **p < 0.001, ***p < 0.0001.

One of the main aims of redefining the model used to estimate the separation threshold is to expand the application range of inertial focusing techniques for samples of different densities, shapes, and sizes. Here, we investigated the accuracy of all three threshold models for irregularly shaped samples using polystyrene (iPS) particles of various shapes and sizes (< 100 μm). Through actual experiments, we obtained the recovery rates of 15 μm, 16.5 μm 21 μm iPS samples (± 2 μm) in outlets corresponding to the thresholds of TM1, TM2, and TM3. The hPRRs of 21 μm, 16.5 μm and 15 μm iPS particles were 41 ± 4.8% (outlet 3), 31.2 ± 10% (outlet 4) and 27.6 ± 4.9% (outlet 3) (Fig. 4). Then, we evaluated whether TM1 will underestimate the threshold by obtaining the PRR of the larger iPS particles (25 ± 2 μm). The hPRR of 25 μm iPS samples (41.7 ± 3.4%) was similar to the hPRR of 21 μm iPS samples. Therefore, the threshold of iPS was likely to be saturated around 21 μm, and TM1 was still a better model for iPS samples.

### Evaluation of all models with irregular high-density particles

**Figure 5:**
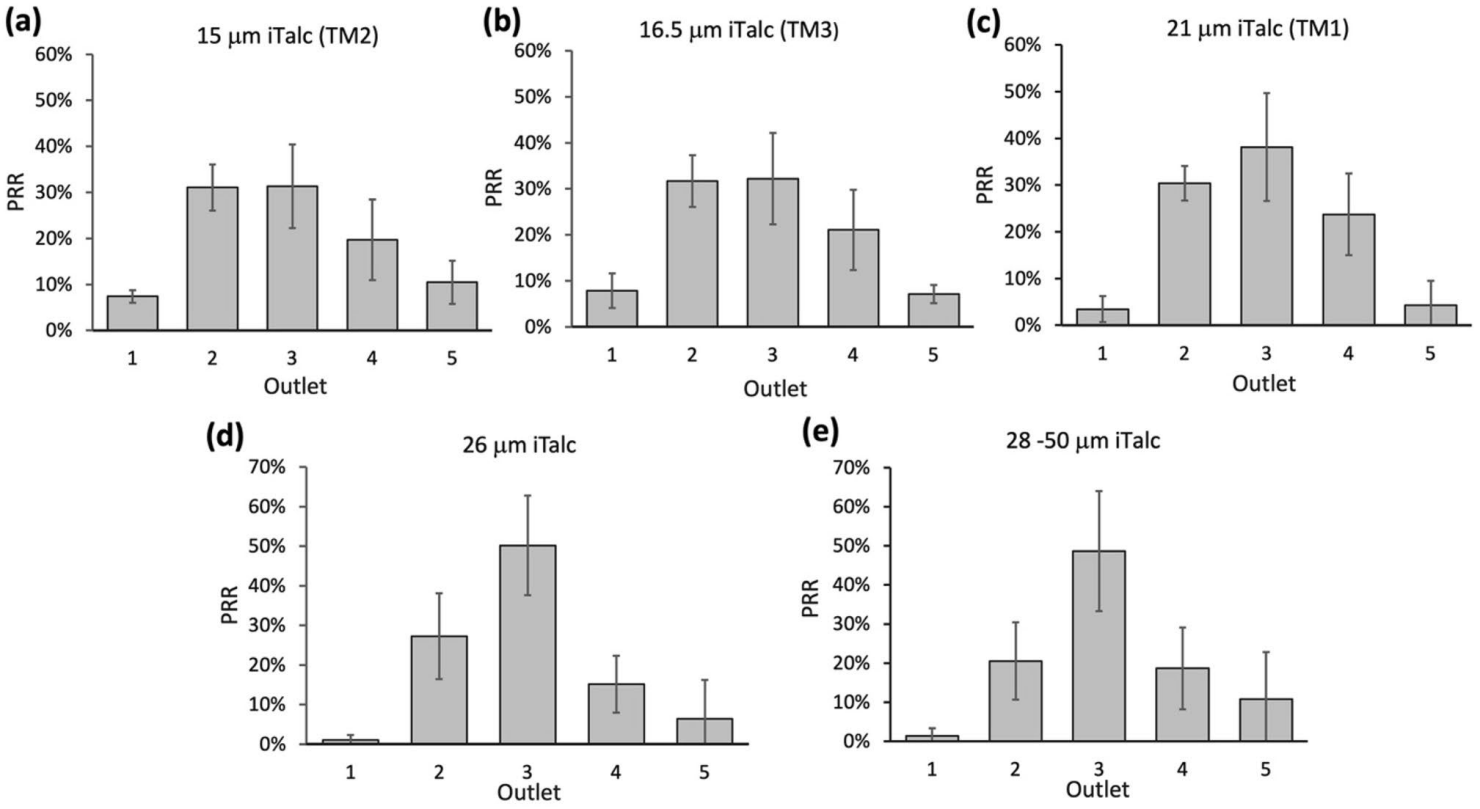
Recovery rate of irregular talcum (iTalc) particles of (**a**) 15 μm, (**b**) 16.5 μm, (**c**) 21 μm, (**d**) 26 μm and (**e**) 28–50 μm (± 2 μm) diameters. As the highest recovery rate of 26 μm iTalc (50.6%) was higher than that of 21 μm (38%), and very close to that of 28–50 μm (48.6%), 26 μm was determined as the threshold for iTalc. Data were shown as mean ± SD of three independent experiments.

The focus position of particles within the channels depends on the particle density^12^. However, since inertial microfluidics research is mainly designed for biomedical applications, low-density particles such as polystyrene microbeads ($\sim 1.05$ g/ml) were often used because their densities are similar to that of biological samples. In recent years, scientists are now interested in exploring the use of inertial microfluidics for higher density targets. Hence we used irregular talcum (iTalc) particles ($\sim 2.75$ g/ml) to investigate how particle density could affect the accuracy of the existing threshold models. It could be seen from the results that the hPRRs of 21 μm, 15 μm and 16.5 μm iTalc particles at outlet 3 were 38 ± 11.5%, 31.3 ± 9.1%, and 32.2 ± 9.9%, respectively (Fig. 5). These low PRRs of respective diameters were probably due to the high density of talcum.

According to Eqs. (3) and (6), *F*_*L*_ and *F*_*D*_ are independent of the change in particle density, and thus an increase of particle density might not influence the balance of *F*_*L*_ and *F*_*D*_. However, in inertial microfluidics, apart from the *F*_*L*_ and *F*_*D*_, the particles migrating in the curved channel were also subjected to the centrifugal force *F*_*c*_ which is given by:

9 $${F}_{C}=\frac{\left({\rho }_{p}-{\rho }_{f}\right)\pi {V}^{2}{a}^{3}}{6r}$$

where ${\rho }_{p}$ and ${\rho }_{f}$ are the particle and fluid densities, respectively, *V* is the tangential velocity of the particles, and *r* is the radius of the particle trajectory^10^. In most studies, *F*_*c*_ is usually ignored for proximate densities of particle and fluid medium (${\rho }_{p}-{\rho }_{f}$)^12^. For regular and non-regular PS samples, since the densities of PS ($\sim 1.05$ g/ml) and water ($\sim 1$ g/ml) are very similar, *F*_*c*_ acting on microbeads is negligible, and thus particle focusing depends on the balance of *F*_*L*_ and *F*_*D*_ (*F*_*L*_ = *F*_*D*_). However, for higher density particles such as talcum, the *F*_*c*_ acting on the particles would be higher (keeping other parameters unchanged), and thus *F*_*c*_ became non-negligible. When the particles were subjected to Dean vortices in the inner cross-section, *F*_*c*_ acted in the direction opposite to *F*_*L*_, and therefore a larger *F*_*L*_ was required to balance the additional *F*_*c*_ and to focus the particles of higher density (*F*_*L*_ = *F*_*D*_ + *F*_*c*_). Hence, the deviation of PRRs values from TM1 estimation strongly suggested the need for a more precise model for high-density targets.

### Modification of TM1 for irregular high-density particles

Since larger particles will experience a larger *F*_*L*_ in the system, the threshold for iTalc particles was expected to be larger compared to that of iPS particles. In order to derive a more precise model for samples with varying densities, we obtained the PRR of the larger iTalc particles (26 ± 2 μm) at each outlet. The results showed that the highest PRR of 26 ± 2 μm iTalc particles (50.2 ± 12.6%) found at outlet 3 was significantly higher than that of 21 μm (38 ± 11.5%) (Fig. 5d). This result was in line with our expectation that the larger *F*_*L*_ induced by larger particles would balance the additional *F*_*c*_. Then, by analyzing the PRR of iTalc particles larger than 26 ± 2 μm (28–50 μm), we assessed whether the threshold was saturated at 26 ± 2 μm. The hPRR for larger iTalc particles at outlet 3 was found to be 48.6 ± 15.3%, which was very similar to the PRR of the smaller iTalc particles (26 ± 2 μm) (Fig. 5e). Therefore, the threshold for high-density particles (iTalc) was probably saturated around 26 μm.

Similar to the result of iPS particles, a slight increase in size did not increase the highest PRR, despite the larger *F*_*L*_ experienced by larger particles. This could be due to the presence of other opposite forces, *F*_*c*_ and *F*_*D,*_ which would also be increased in the system (*F*_*c*_ $\propto {a}^{3}$ and *F*_*D*_ $\propto a$). Therefore, there was a saturation point at which the focusing effect did not increase as the particle dimension increased. To allow estimation of PRRs for samples with varying densities, we thus derived a model (TM4) as:

10 $$\frac{a}{D} \ge 0.07c$$

where *c* was a density coefficient greater than or equal to unity (c $\ge 1).$ The value of *c* increased with the density of sample, for example, for polystyrene particles ($c =1; \sim 1.05$ g/ml) and for talcum particles ($c = 1.215; \sim 2.75$ g/ml). Since the c value is equal to 1 for PS, and 1.215 for Talc, substituting above c values to TM4, the thresholds for iPS and iTalc particles were calculated to be 21 μm and 26 μm, corresponding to the highest PRRs obtained previously (Figs. 4 and 5).

### Applicability of TM4 for irregular particles of different densities

**Figure 6:**
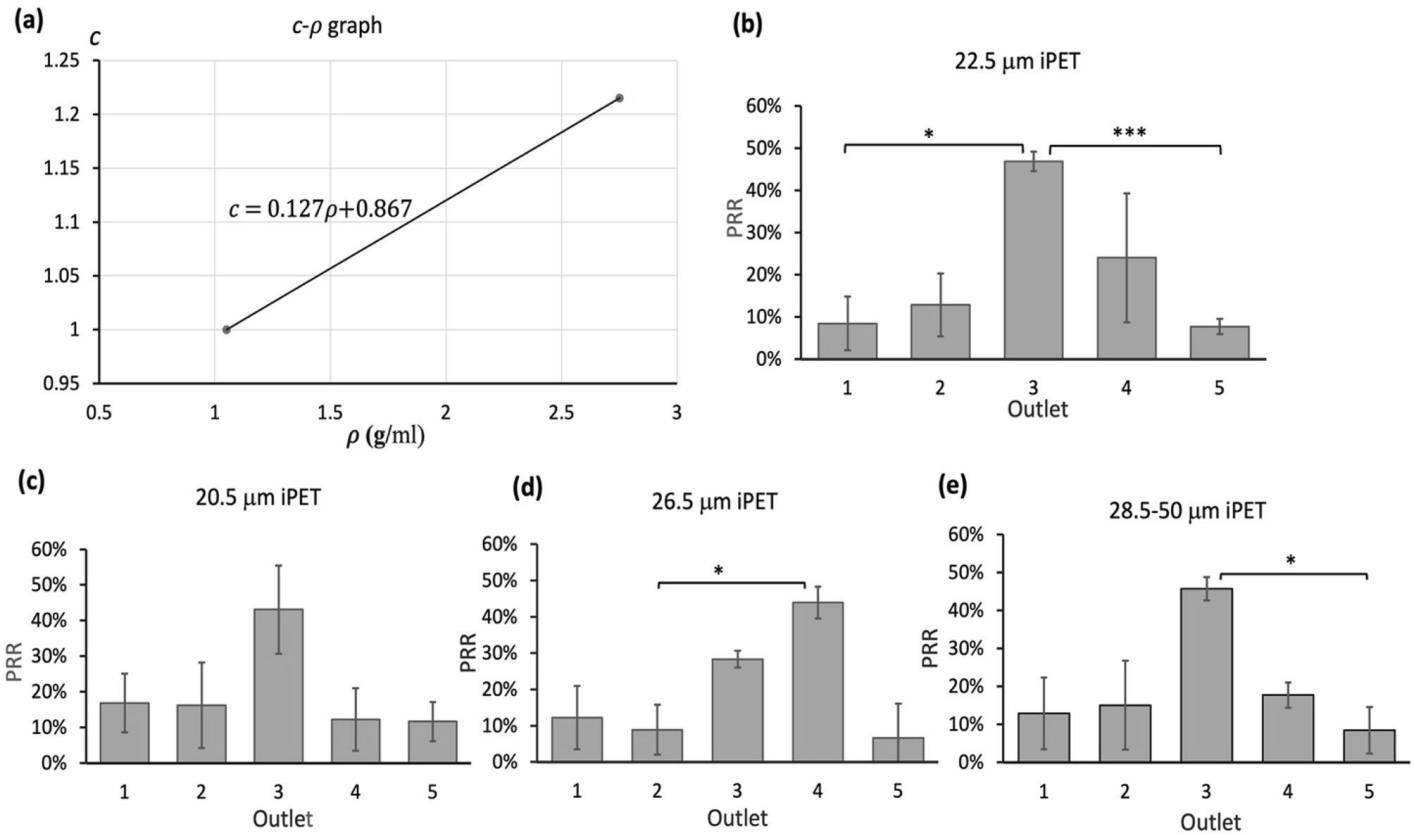
The proposed linear correlation between density coefficient, particle density, and the recovery rate of irregular polyethylene terephthalate (iPET) of various diameters. (**a**) The graph illustrates that *c* linearly increased with $\rho$ within the range of 1.05 g/ml to 2.75 g/ml. The PRRs of (**b**) 22.5 ± 2 μm, (**c**) 20.5 ± 2 μm, (**d**) 26.5 ± 2 μm, and (**e**) 28.5–50 μm were indicated respectively. Since the hPRR of 22.5 μm was similar to 26.5 μm but higher than that of 20.5 μm, 22.5 μm was determined as the threshold for iPET. Data were shown as mean ± SD of three independent experiments. *p < 0.01, **p < 0.001, ***p < 0.0001.

**Figure 7:**
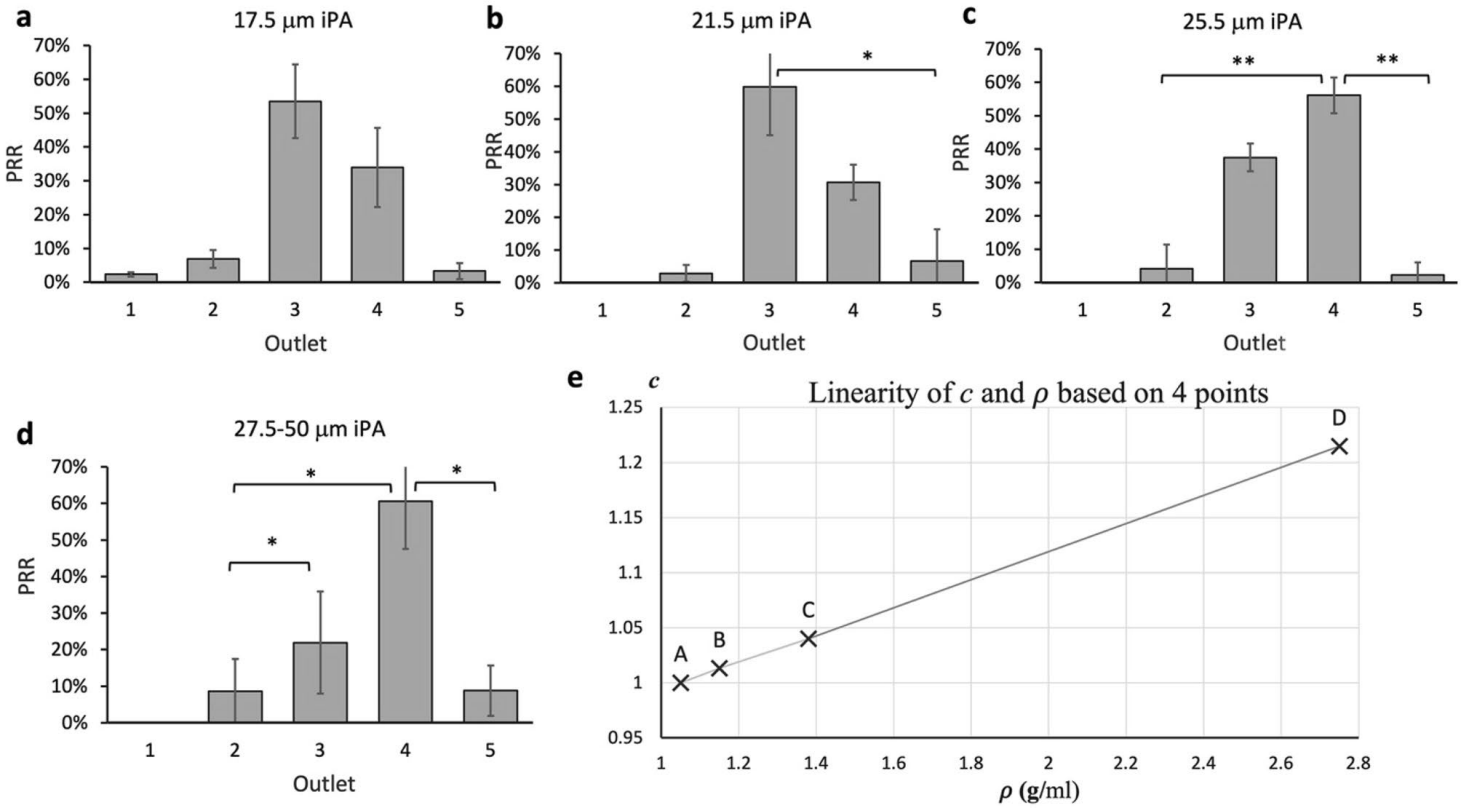
Recovery rate of irregular polyamide (iPA) particles of (**a**) 17.5 ± 2 μm, (**b**) 21.5 ± 2 μm, (**c**) 25.5 ± 2 μm, (**d**) 27.5–50 μm diameters. As the highest recovery rate of 21.5 ± 2 μm iPA (59.8%) was higher than that of 17.5 ± 2 μm (52.1%), and very close to that of 25.5 ± 2 μm (55.7%), 21.5 μm was determined as the threshold for iPA. Data were shown as mean ± SD of three independent experiments. *p < 0.01, **p < 0.001, ***p < 0.0001. (**e**) The linearity of density coefficient and particle density. Points A, B, C, and D corresponded to the experimental data obtained with iPS, iPA, iPET, and iTalc particles, respectively.

In order to study whether TM4 could be applied to particles of different densities, we hypothesized that the density coefficient *c* of TM4 would increase linearly with particle density, from 1.05 g/ml to 2.75 g/ml (Fig. 6a), whereby the proposed linear correlation can be modelled by:

11 $$c=0.127\rho +0.867$$

where *c* is the density coefficient, and $\rho$ is the particle density, respectively. We aimed to verify this linear correlation using irregular polyethylene terephthalate (iPET) ($\sim 1.38$ g/ml) and polyamide (iPA) ($\sim 1.15$ g/ml). According to Eq. (11), the density coefficient (*c*) of iPET was around 1.04. Based on TM4, the threshold for iPET was approximately 22.5 μm. We then obtained the PRRs of iPET particles of various sizes (22.5 ± 2 μm, 26.5 ± 2 μm, 20.5 ± 2 μm, and 28.5–50 μm). We found that the highest recovery rates of 22.5 μm, 20.5 μm, and 26.5 μm iPET particles were around 46.9 ± 2.3%, 42.1 ± 12%, 43.9 ± 4.3% and 46.2 ± 5.1% respectively (Fig. 6b–e). As the highest PRR of 22.5 μm particles was higher than that of 20.5 μm particles and close to 26.5 μm and 28.5–50 μm iPA particles, 22.5 μm was determined as the threshold for iPET particles. Then, iPA particles were further used to validate the linearity of the Eq. (11). By substituting c = 1.013 to TM4 (c = 1.013 for 1.15 g/ml), the threshold for iPA was determined as approximately 21.5 μm. To investigate if 21.5 ± 2 μm was the threshold of iPA particles via experiments, we obtained the PRRs of 17.5 ± 2 μm, 21.5 ± 2 μm, 25.5 ± 2 μm and 27.5–50 μm dimensions respectively. The results (Fig. 7a-d) showed that the threshold for iPA was around 21.5 μm (60% hPRR at outlet 3) because the hPRR of 21.5 μm iPA was higher than that of 17.5 ± 2 μm and similar to that of 25.5 ± 2 μm. Therefore, the linearity was validated (Fig. 7e).

Overall, we derived an improved model (TM4) to estimate the separation efficiency of samples with varying densities by incorporating the factor of particle density. Our studies demonstrated that TM1 was only suitable for the low-density particle (1.05 g/ml), while TM4 was applicable for samples with a broader range of densities, including high-density samples (2.75 g/ml). The linear correlation between *c* and $\rho$ makes TM4 more applicable to samples of different densities. For example, since the value of *c* is 1 in TM4 for PS particles, TM4 will be exactly equivalent to TM1, which was the most accurate for PS particles, and thus TM4 retained the same accuracy as compared to TM1 for low-density samples. For intermediate-density and high-density particles (e.g., PET and Talc), since the centrifugal force acting on them was non-negligible, the parameter was considered in the derivation of TM4 by introducing a density coefficient *c*, which was customized for particles of different densities. Specifically, the threshold of suitable density range for TM4 was between 1.05 to 2.75 g/ml. We hope these studies can also provide a systematic approach for future efforts to derive an optimal prediction model for a wider range of biological samples.

## Discussion

Some parameters essential to channel design, such as *C*_*L*_ and *U*_*D*_ in Eq. (3) and (5) can only be estimated through experimental data^12^. These equations can be modified by utilizing the size-dependent nature of *R*_*f*_ (*R*_*f*_ = *F*_*L*_*/F*_*D*_ and *R*_*f*_ ∝ *a*^3^). However, there is a lack of standardisation of the threshold model used, leading to a significant discrepancy in threshold prediction. In addition, current models were developed using low-density samples ($\sim 1.05$ g/ml) and may not be applicable to samples of higher density. We believe that the applications of inertial microfluidics will be extended to the use of high-density particles in the foreseeable future. One potential application is to facilitate the monitoring of heavy metals in drinking water. These high-density metals (such as Ag, Cu, Ni, and Cr) are carcinogens present in very low concentrations. Nonetheless, current point-of-care technologies for monitoring metals are subjected to high limits of detection (LOD), of which the LOD values are higher than the maximum allowable limit suggested by WHO^24^. Therefore, an improved threshold model incorporating sample density for point-of-care inertial microfluidic biosensors can be useful for the detection or removal of toxic metals from drinking water.

Overestimation of the threshold, even as small as a few microns, will also significantly affect the design of a microfluidic device and prevent the optimal separation of the subpopulations of cells. For example, the diameter of white blood cells ($\sim$ 9.5 μm) is similar to that of red blood cells ($\sim$ 7 μm). Here, we evaluated the accuracy of the existing models (TM1 and TM2) and derived an improved model (TM4) to estimate the separation efficiency of samples with varying densities by incorporating the factor of particle density. TM4 provides a more precise estimation of the separation efficiency of samples with varied densities and will aid researchers in their design of microchannels for particle separation based on the inertial focusing effect.

Similar to TM1 and TM2, it should be highlighted that TM4 is only applicable to channels with a rectangular cross-section. Interestingly, the previous threshold models did not consider geometric considerations, and both TM1 and TM2 were derived based on rectangular cross-section channels. Since the focus of this manuscript is to expand on existing threshold models (TM1 and TM2) and incorporate the component of sample density for inertial microfluidics, the standard rectangular cross-section design was used for our simulation and experimentations. Our preliminary studies (Fig. S2) suggested that all current threshold models (including TM4) were indeed not applicable for microfluidic channels with varied geometric designs (e.g., trapezoidal). Future efforts should be directed towards the modification of existing models for different geometric shapes.

Other factors can also influence the sorting efficiency of particles. Previous studies have also emphasized that unless the asymmetry is large, the shape of the particle has only a small effect on inertial focusing, although the relevant mechanism was not clarified in the literature^25^. In order to show whether there were some PS powders with great asymmetry, a scatter diagram of the circularity distribution of iPS powders was generated (Fig. S3). Since the scattering points were uniformly distributed from 0.1 to 1, the shape of the particles was highly diverse. This implied that a certain portion of particles could have a higher degree of asymmetry. As previous research on the correlation between the particle shape and the focus position was carried out with straight channels, further studies will be required to consider how Dean resistance is affected by the shape of the particles.

The experimental results of the microbeads were different from that of the simulated runs. For example, experimental results showed that outlet 4 had a higher PRR of 21 μm PS microbeads, which could be due to the stronger *F*_*L*_ experienced. On the contrary, the 16.5 μm microbeads were less focused in outlet 3, possibly due to a moderate *F*_*L*_ acting on the microbeads^26^. The shift of the equilibrium position from outlet 4 to 3 in experimental results could also be due to differential positioning depending on the nature of *F*_*L*_. Particles circulating along with Dean vortices at the inner section would experience larger *F*_*L*_ at the proximate center location than at proximate inner channel wall^7^. Compared with 21 μm microbeads, 15 μm and 16.5 μm microbeads could experience sufficient *F*_*L*_ to balance *F*_*D*_ when they were located in a lateral position closer to the center of the channel. In this study, COMSOL was only a means to provide a rough estimation, as the accuracy of COMSOL was also influenced by many factors, such as the number of mesh elements. Increasing the number of elements generally led to higher accuracy, but the demand for computation power was also higher. In this study, the number of mesh elements was standardised to around 37,000. Despite these limitations, we demonstrated that the outcomes of the numerical simulation were close to our actual experiments of 15 μm and 21 μm microspheres.

Biological samples are often heterogeneous, especially when clinical samples are involved. Prior studies on the detection of mesenchymal cancer cells from bladder wash urine and blood samples had demonstrated the fluctuations in target cell retrieval in clinical samples^23^. Here, we evaluated the performance of different models using particles of different size, shape, and density. Our result demonstrated that TM1 was a more accurate model for low-density particles. However, PPRs of higher density samples deviated from expected values. We thus modified TM1 by considering the effect of the additional centrifugal force *F*_*c*_ acting on high-density particles in the curvilinear channel. The derived model (TM4) comprises of a density coefficient *c* such that the threshold obtained fits experimental values. The robustness of TM4 for samples of varying densities (1.05 g/ml $\le \rho \le$ 2.75 g/ml) was confirmed with intermediate-density particles (PET and PA). In addition, TM4 was also as accurate as TM1 for low-density particles (Table S3).

Future analysis should cover other parameters that could influence the threshold, such as changes in channel aspect ratio, channel radius (*R*), and cross-section dimensions (same aspect ratio), which were reported to affect inertial focusing^27^. For example, apart from shape and density, particle deformability is one of the factors affecting the inertial effect owing to an additional life force experienced by non-rigid particles^28^. Similar to other inertial studies using polymeric particles (usually PS), all particles were assumed to be rigid because of their relatively high elasticity ($\sim 2-4 GPa$). However, the mechanical characterization of cells is similar to a drop of liquid enclosed by a membrane exhibiting low elasticity^29^. For example, the elastic modulus of mammary cells (MCF10A) and breast cancer cells (MCF7) widely used for studying breast cancer were around 440 Pa and 300 Pa, respectively^30^, while the stiffness of healthy leukocytes was even lower (10–60 Pa)^31^. Based on differences in deformability, isolation of more deformable MCF7 and healthy leukocytes from less deformable MCF10A and immature leukocytes had been reported in previous studies despite their similarity in sizes^31,32^.

Overall, we demonstrated that TM1 remained accurate for regular and non-regular low-density particles (e.g., PS). However, a modified model TM4, which incorporated the density coefficient *c* (TM4), was more suitable for samples with varied densities, especially those with high-density particles (e.g., Talc). The linear correlation of density coefficient *c* and particle density $\rho$ was also validated. These findings are highly applicable to applications where the density of targeted particles is high or heterogeneous.

## Methods

### Device feature and fabrication

The spiral device with a rectangular cross-section (the main device in this study) consisted of a 10-loop curvilinear channel with 300 μm spacing between loop interval. The width and height of the channel were 500 μm and 220 μm, respectively. The device had 1 inlet and 5 outlets. The end of the 500 μm wide channel was evenly split into five outlets, each of which had 100 μm width. Spiral devices with trapezoidal cross-section consisted of an 8-loop curvilinear channel with 300 μm spacing between loop interval. The width of the channel was 600 μm, while the inner and outer heights were 80 μm and 130 μm, respectively. The device had 1 inlet and 2 outlets. The 600 μm wide channel was evenly split into two outlets.

Microdevices were fabricated using standard soft lithography from an aluminum master mold. Briefly, polydimethylsiloxane (PDMS) was prepared by mixing a 10:1 ratio of base and curing agent (Dow, Germany). The prepared PDMS was degassed in degasser and poured onto the master mold. The mold filled with prepared PDMS was baked at 70 °C oven for 2 h. After baking, the solidified PDMS was peeled off from the mold carefully. The locations for inlet and outlets were punched using a 1.5 mm diameter puncher (Integra, USA). The punched PDMS device was bonded to a flat solidified PDMS layer to complete the channels in a plasma machine. After plasma treatment for 3 min, the bonded PDMS device was placed in a 70 °C oven for 30 min to strengthen the bonding. Finally, 15 cm and 6 cm long sections of flexible plastic tubing (Tygon, USA) were inserted into the device inlet and outlets, respectively.

#### COMSOL flow simulation

To evaluate the particle focusing of different sizes for respective threshold models. COMSOL Multiphysics software was used to simulate the inertial focusing on our existing microdevice. The three-dimensional (3D) image of the microdevice was created by AutoCAD in STL format (Fig. 1a). All dimensions were the same, except for the shorter length of the branch at all outlets (to save computation time). The properties of water were applied to systematic fluid for simulation (ρ $=1000\,kg {m}^{-3}; \mu ={10}^{-3}\,kg {m}^{-1}{s}^{-1}$). *The laminar flow (spf)* module with incompressible flow property was used in the system. $F$ low velocity was set at the inlet, while the fixed pressure boundary condition was set at all outlets. Using these perimeters, a flow magnitude profile for the system was simulated (Fig. 1b).

Based on the simulation of the flow profile, *particle tracing for the fluid flow (fpt)* module was chosen to analyse the motions of particle trajectories. The drag force, wall-induced life force, and shear gradient life force parameters were applied to the system. Interactions between particles were ignored as the diluted sample was often used in the inertial microfluidic application. Solid particles ($1.05$ g/ml) was used to model spherical polystyrene (PS) microbeads. Each set of samples consisted of 3 particles of the same size evenly distributed at the inlet to initiate simulation (Fig. 1a). The simulation time for each set of particles was 2 s with 0.01 s time step for tracing particle trajectories. The '*freeze*' condition was applied at the end of outlets to ensure all particles within the boundaries. The number of particles freezing at the end of boundaries was counted.

#### Flow rate optimization

For the inertial microfluidic system, the Reynolds number (*Re*) is required to be within the range of 1 to 100 (~ 1 < *Re* <  ~ 100), which corresponds to the volumetric flow rates approximately from 0.021 ml/min to 2.1 ml/min. Since a higher flow rate was preferable, different flow rates (1.5 ml/min, 1.7 ml/min, 1.9 ml/min, and 2.1 ml/min) were used to process 21 μm PS regular microbeads in the system. 21 μm spherical microbeads were used as the particle size was above the thresholds of both TM1 and TM2, which indicated that 21 μm PS microbeads should be well-focused in the device. We confirmed that based on the highest PRR (91.7% PRR) obtained under this parameter, the optimal flow rate is 1.7 ml/min (Fig. S4). Although the PRR derived from the flow rate of 1.9 ml/min suggested that this parameter could also be used, it was close to the limit of Reynold's number applicable to inertial focusing based microfluidics, and thus only 1.7 ml/min was used for subsequent experiments.

#### Preparation of irregular particles

iTalc (baby powder) was commercially available (Johnson & Johnson China Ltd, China). For iPS, iPET, and iPA powders, they were prepared using a Retsch CryoMill cryogenic grinder (Haan, Germany). After a 7-min precooling stage with five shakes per second, the grinding stage was used for 1.5 min with 25 shakes per second in the presence of liquid nitrogen. The crushed microplastic powders (< 100 μm) were then collected through a 100 μm filter. The powders were mixed with DI water for experimentation in a biological safety cabinet to minimize potential contamination.

### Particle sorting experiments

1 μl microbead suspension was mixed well with 1 ml deionized water and transferred to 10 cc syringe. Another 10 cc syringe was prepared by injecting 2 ml deionized water and was loaded to a syringe pump. The inlet and outlets of the microdevice were connected to the syringe and five centrifugal tubes using plastic tubing. The device and centrifugal tubes were arranged accordingly (Fig. 1c). The syringe pump was run at 1.7 ml/min for 10 s. The loaded syringe was replaced by another syringe with microbead suspension. The syringe pump (New Era Pump Systems Inc., USA) was run at 1.7 ml/min for 20 s. The sample collected at centrifugal tubes was then transferred to a 24-well plate (SPL Life Science, Korea). Each well with microbead suspension was mixed well with an additional 100 μl deionized water. The 24-well plate was placed at rest for 30 min to settle down the particle suspension. The number of regular particles in each well was counted under an optical microscope (Nikon, Japan) (Fig. S5). All experiments were carried out in triplicates.

For irregular particles, all images captured by the optical microscope (Fig. S6) were analysed by ImageJ. Briefly, images were converted to 8 bits with suitable threshold levels to customize the particle counting function. The adjusted images were carefully compared to their original images to ensure that the threshold selected was optimal. Their circular diameters were measured with ImageJ and Excel. Briefly, we first obtained the boundary area (A) of each particle by ImageJ. Then, the circular diameter (d) was calculated by d = √ (4*A/π) using Excel. Due to the high variation of sample sizes, the upper and lower ranges of two microns (± 2 μm) were included for the measurement of the specific dimension.

## Supplementary information

Supplementary Information.

## Abbreviations

TM: Threshold model
DLD: Deterministic lateral displacement
PFF: Pinched flow fractionation
PRR: Particle recovery rate
hPRR: Highest particle recovery rate
PS: Polystyrene
iPS: Irregular polystyrene
Talc: Talcum
iTalc: Irregular talcum
PET: Polyethylene terephthalate
iPET: Irregular polyethylene terephthalate
PA: Polyamide
iPA: Irregular polyamide
LOD: Limits of detection
