# Supplementary material for: A density-based threshold model for evaluating the separation of particles in heterogeneous mixtures with curvilinear microfluidic channels
Source: Sci Rep. 2020 Nov 4;10:18984. doi: 10.1038/s41598-020-75878-w (PMC7643107; doi:10.1038/s41598-020-75878-w)
Supplement: Supplementary file 1 — Supplementary Information. [file 41598_2020_75878_MOESM1_ESM.docx]

**Supplementary Materials**

A density-based threshold model for evaluating the separation of particles in heterogeneous mixtures with curvilinear microfluidic channels

Chun Kwan Chen ^1^, Bee Luan Khoo ^1,*^

^1^ Department of Biomedical Engineering, City University of Hong Kong, Hong Kong, China

* Contact:

Bee Luan Khoo (blkhoo@cityu.edu.hk)

City University of Hong Kong

83 Tat Chee Avenue, Kowloon, Hong Kong, China

Orcid ID: 0000-0003-1100-9994

**Figures**

**
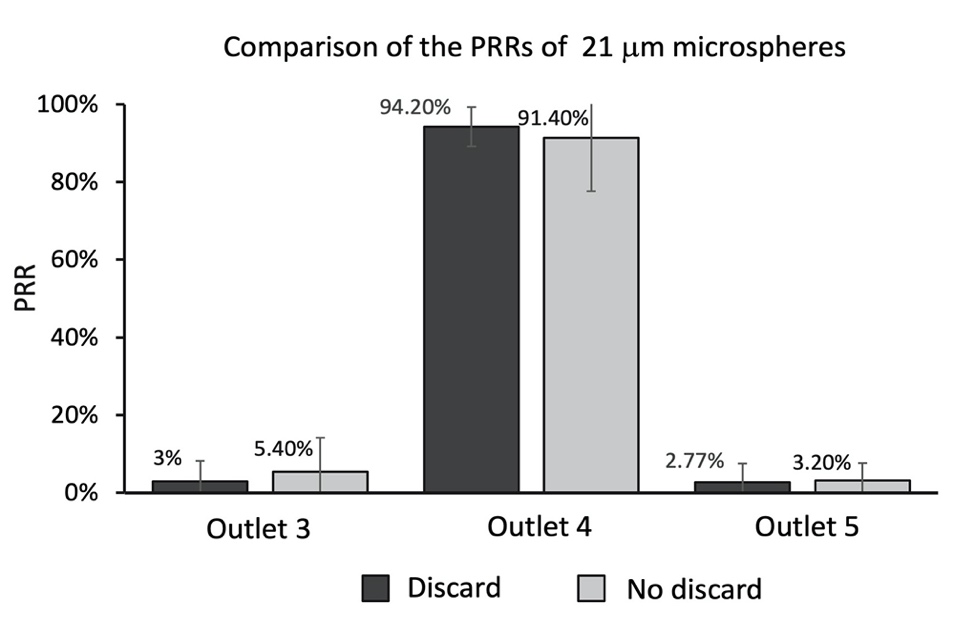
**

**Fig. S1.** Comparison of the PRRs of 21 μm microspheres with and without discarding the sample within the first 5 seconds. The results showed that by discarding the sample from the first 5 seconds, the PRR of the target outlet (outlet 4) increased (94.2%). The results supported the hypothesis that the particles found in outlets 3 and 5 were the result of unfocused streamlines in the first few seconds of flow.

**
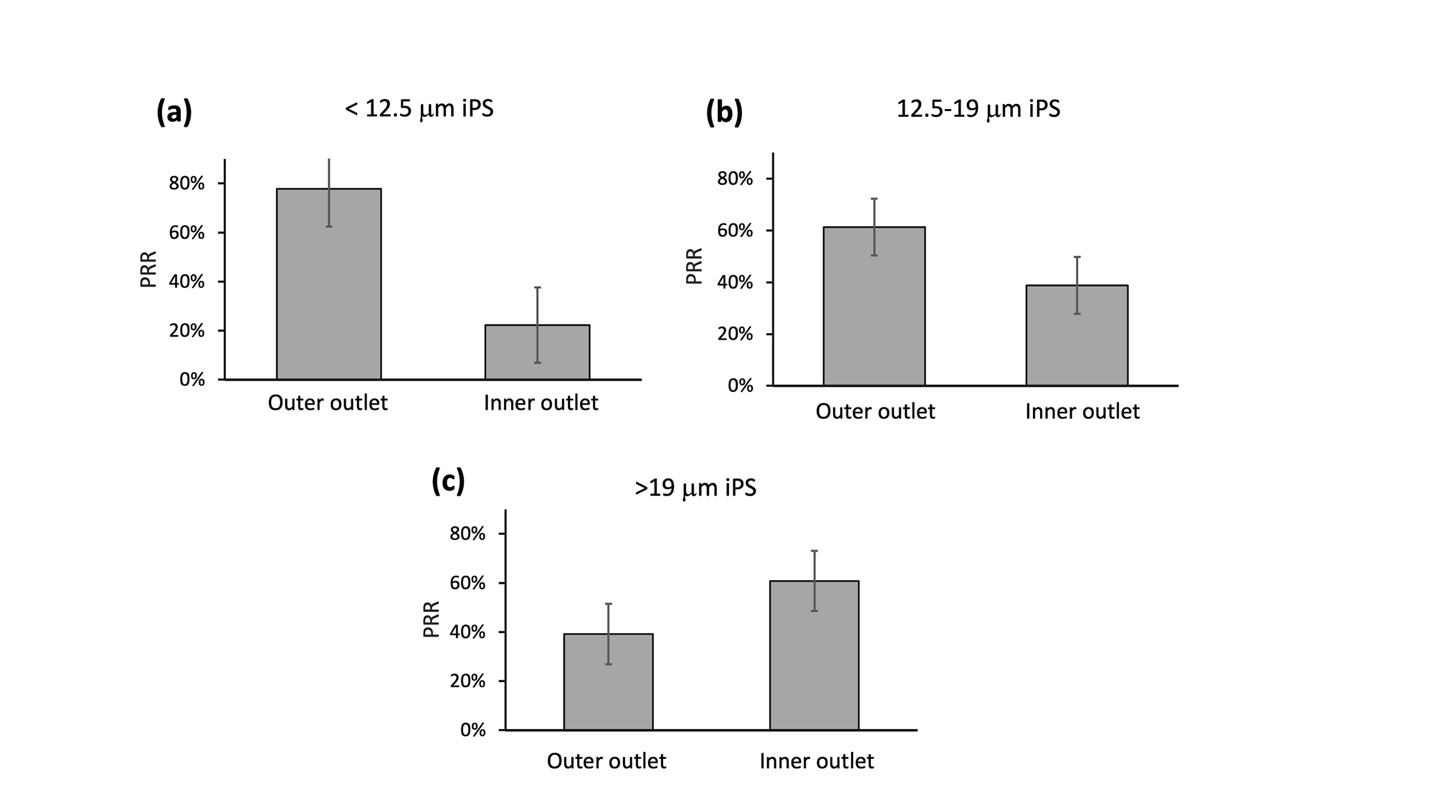
Fig. S2.** The particle recovery rates (PRRs) of iPS particles obtained by spiral devices with a trapezoidal cross-section. According to TM4, the calculated threshold for iPS was around 12.5 μm. We obtained the PRRs of (a) < 12.5 μm, (b) 12.5 – 19 μm and (c) > 19 μm iPS particles respectively. Since the PRR of 12.5 – 19 μm iPS at the inner outlet was not higher than that at the outer outlet, the calculated threshold of 12.5 μm was inaccurate, indicating that both TM1 and TM4 were not applicable to trapezoidal cross-sectional channels. Data were shown as mean ± SD of three independent experiments.

**
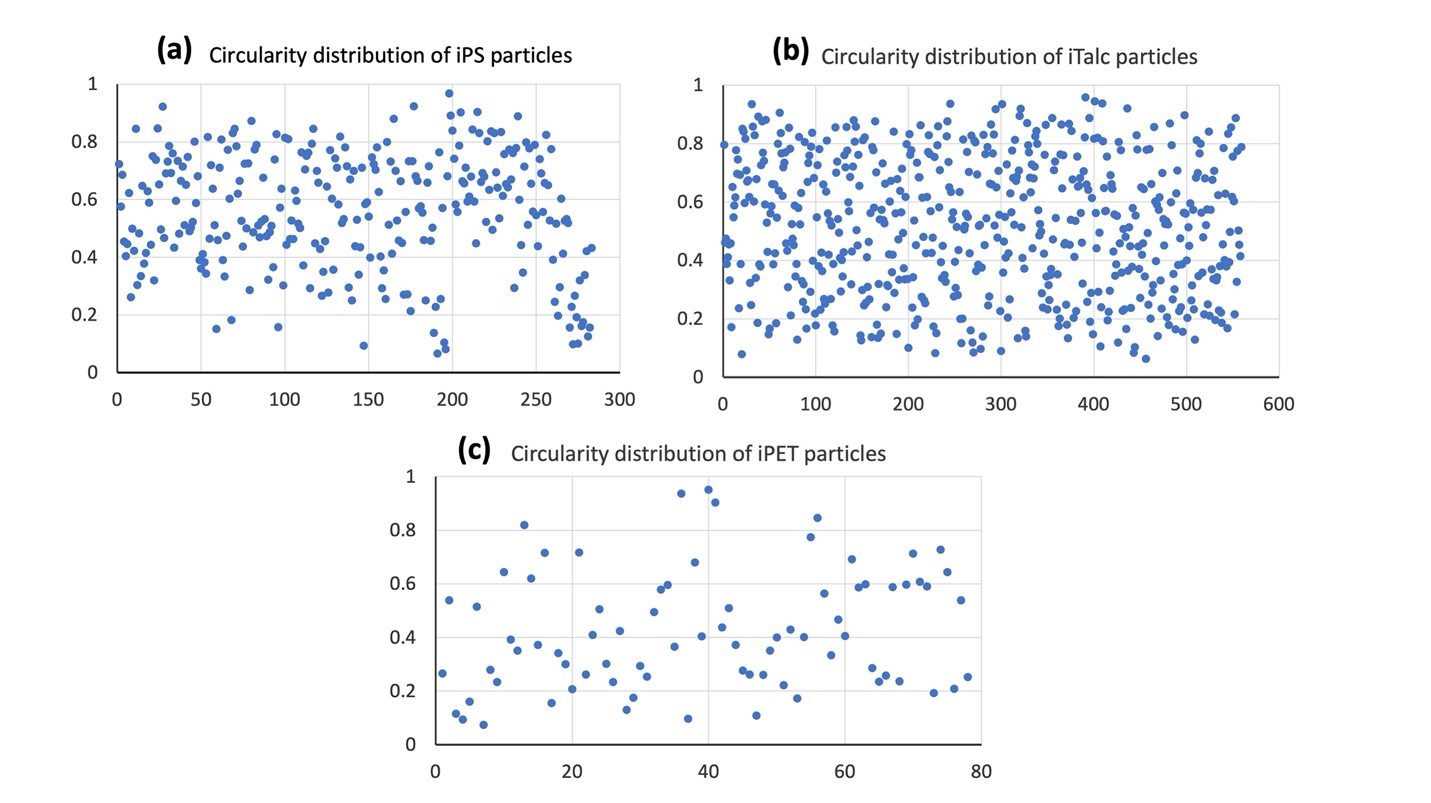
** **Fig. S3.** The circularity distribution profiles of irregular (a) PS, (b) Talc, and (c) PET samples. The x-axis corresponded to the number of particles. Circularity (y-axis) approaching 1 indicated that the shape of the particles became more circular.


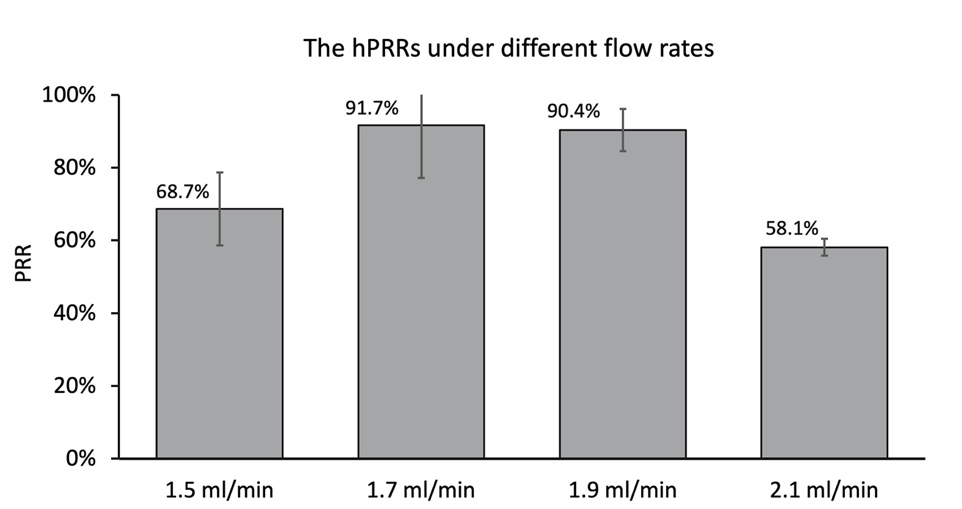


**Fig. S4.** Bar chart demonstrating the highest PRRs of 21 μm spherical particles at flow rates of 1.5 ml/min, 1.7 ml/min, 1.9 ml/min, and 2.1 ml/min, respectively. Data were shown as mean ± SD of three independent experiments.

**
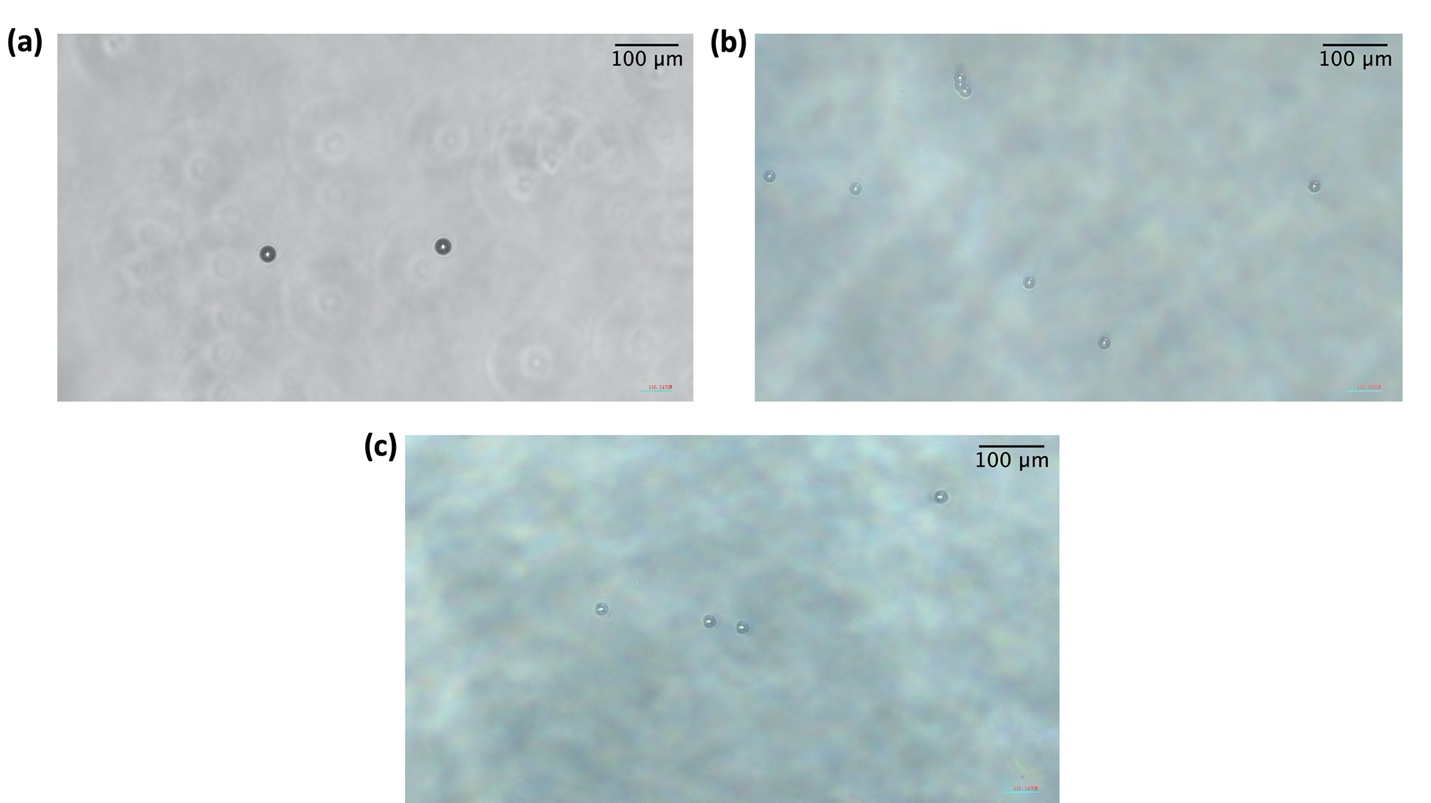
Fig. S5.** Representative images of regular (a) 21 μm, (b) 15 μm and (c) 16.5 μm PS microbeads under the optical microscope. Scale bar is 100 μm.

**
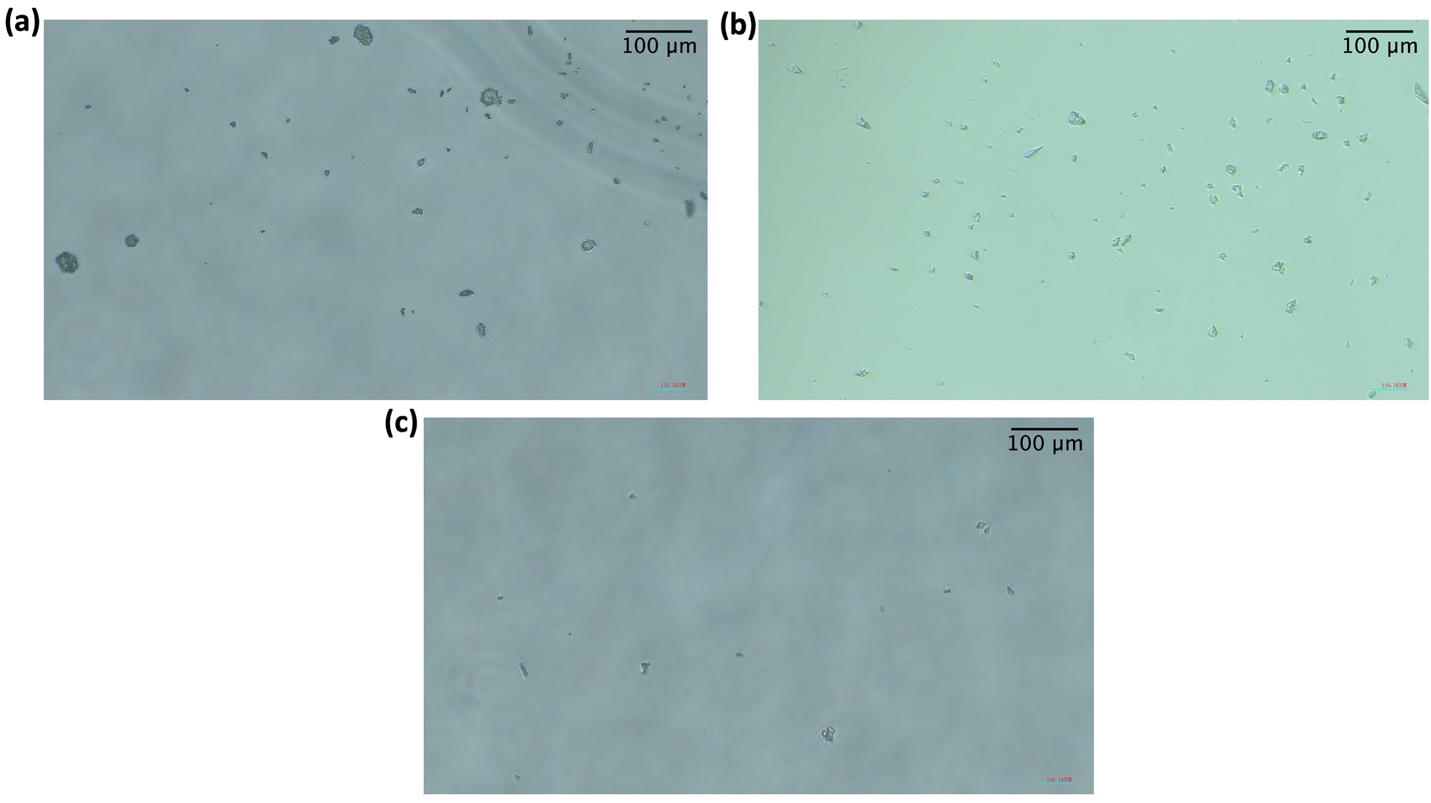
Fig. S6.** Representative images of irregular (a) PS, (b) Talc, and (c) PET particles under an optical microscope. Scale bar is 100 μm.

**Tables**

**Table S1.** Microfluidic technologies for label-free separation of particles.

| Techniques. | Separation criteria | Target | Throughput | Sensitivity | Merits | Demerits | Ref. |
| --- | --- | --- | --- | --- | --- | --- | --- |
| Active | | | | | | | |
| Acoustophoresis | Size, density, compressibility | Red blood cells | 80 μl/min  (Whole blood) | 100%  (Red blood cells) | High sensitivity,  fairly high throughput | Alteration of cell phenotype reported | ^1,2^ |
| Dielectrophoresis | Polarizability, size | Red blood cells | < 6 μl/min  (Whole blood) | >99%  (Red blood cells) | High sensitivity,  high resolution ($\sim$0.5 μm) | possible sample damage,  low throughput | ^2,3^ |
| Passive | | | | | | | |
| Gravitation separation | Size | Microbeads  (1-20 μm) | $\sim$17 μl/min  (Microbeads) | $\sim$99.7%  (Microbeads) | High sensitivity | Low throughput and resolution ($\sim$17 μm),  complicated fabrication | ^2,4^ |
| DLD | Size, deformability | Red blood cells | $\sim$5.8 μl/min  (50% diluted blood) | 99.99%  (Red blood cells) | High sensitivity,  nanoscale resolution ($\sim$10 nm) | Low throughput,  complicated device fabrication,  channel clogging | ^5,6^ |
| PFF | Size | Microbeads  (1-5 μm) | $\sim$0.3 μl/min  (Microbeads) | $\sim$99%  (Microbeads) | High sensitivity and resolution ($\sim$1 μm) | Low throughput | ^7,8^ |

**Table S2**. Averaging tests for the derivation of TM3. T_t0_ = PRR of 21 μm; n denotes the n^th^ averaging test.

| n | Ref. range (μm) | Testing value (T_tn_) | Is PRR of T_tn_ different from that of T_tn-1_? | Updated ref. range (μm) |
| --- | --- | --- | --- | --- |
| 1 | $15<T<21$ | 18 μm | No | $15<T<18$ |
| 2 | $15<T<18$ | 16.5 μm | No | $15<T<16.5$ |
| 3 | $15<T<16.5$ | 15.7 μm | Yes | $15.7<T<16.5$ |

**Table S3**. The highest PRRs of samples corresponding to the thresholds of TM1, TM2, TM3, and TM4.

| Model | $\frac{a}{x}\geq0.07$ | Threshold | Regular PS  $(1.05 g/ml$ | Irregular PS  $(1.05 g/ml)$ | Irregular PET  $(1.38 g/ml)$ | Irregular Talc  $(2.75 g/ml)$ |
| --- | --- | --- | --- | --- | --- | --- |
| TM1 | $x=D$ | 21 μm | 91.6% | 41% | 45.2% | 38.1% |
| TM2 | $x=h$ | 15 μm | 52.4% | 31.2% | 23.6% | 31.3% |
| TM3 | $x=\frac{D+3h}{4}$ | 16.5 μm | 77.6% | 27.6% | 33.1% | 32.2% |
| TM4 | $\frac{a}{D}\geq0.07c$ | Varied with *c* | 91.6%  (21 μm) | 41%  (21 μm) | 46.9%  (22.5 μm) | 50.2%  (26 μm) |

**Table S4**. The number of particles in each outlet.

| Sample | Trials | Outlet 1 | Outlet 2 | Outlet 3 | Outlet 4 | Outlet 5 |
| --- | --- | --- | --- | --- | --- | --- |
| 15 μm microbeads | 1 | 0 | 0 | 54 | 49 | 0 |
|  | 2 | 0 | 0 | 4 | 3 | 0 |
|  | 3 | 0 | 0 | 11 | 12 | 0 |
| 16.5 μm microbeads | 1 | 0 | 0 | 187 | 83 | 0 |
|  | 2 | 0 | 0 | 13 | 3 | 0 |
|  | 3 | 0 | 0 | 20 | 4 | 0 |
| 21 μm microbeads | 1 | 0 | 0 | 0 | 14 | 0 |
|  | 2 | 0 | 0 | 3 | 23 | 3 |
|  | 3 | 0 | 0 | 0 | 38 | 0 |
| iPS particles | 1 | 66 | 64 | 130 | 76 | 26 |
|  | 2 | 68 | 90 | 116 | 135 | 56 |
|  | 3 | 67 | 71 | 122 | 51 | 61 |
| iTalc particles | 1 | 55 | 102 | 234 | 109 | 127 |
|  | 2 | 124 | 349 | 338 | 329 | 89 |
|  | 3 | 38 | 92 | 144 | 57 | 53 |
| iPET particles | 1 | 17 | 18 | 18 | 35 | 16 |
|  | 2 | 34 | 38 | 38 | 25 | 15 |
|  | 3 | 10 | 20 | 18 | 11 | 12 |
| iPA particles | 1 | 47 | 133 | 187 | 156 | 48 |
|  | 2 | 37 | 55 | 75 | 30 | 47 |
|  | 3 | 24 | 60 | 164 | 53 | 43 |

References

1 Yu, Z. T., Aw Yong, K. M. & Fu, J. Microfluidic blood cell sorting: now and beyond. *Small* **10**, 1687-1703, doi:10.1002/smll.201302907 (2014).

2 Gossett, D. R. *et al.* Label-free cell separation and sorting in microfluidic systems. *Anal Bioanal Chem* **397**, 3249-3267, doi:10.1007/s00216-010-3721-9 (2010).

3 Vahey, M. D. & Voldman, J. An equilibrium method for continuous-flow cell sorting using dielectrophoresis. *Analytical chemistry* **80**, 3135-3143 (2008).

4 Huh, D. *et al.* Gravity-driven microfluidic particle sorting device with hydrodynamic separation amplification. *Analytical chemistry* **79**, 1369-1376 (2007).

5 Wunsch, B. H. *et al.* Nanoscale lateral displacement arrays for the separation of exosomes and colloids down to 20 nm. *Nature nanotechnology* **11**, 936 (2016).

6 Holmes, D. *et al.* Separation of blood cells with differing deformability using deterministic lateral displacement(dagger). *Interface Focus* **4**, 20140011, doi:10.1098/rsfs.2014.0011 (2014).

7 Takagi, J., Yamada, M., Yasuda, M. & Seki, M. Continuous particle separation in a microchannel having asymmetrically arranged multiple branches. *Lab Chip* **5**, 778-784, doi:10.1039/b501885d (2005).

8 Yamada, M., Nakashima, M. & Seki, M. Pinched flow fractionation: continuous size separation of particles utilizing a laminar flow profile in a pinched microchannel. *Analytical chemistry* **76**, 5465-5471 (2004).
